# Corneal Transplantation for Infectious Keratitis: A Prospective Dutch Registry Study

**DOI:** 10.1097/ICO.0000000000003218

**Published:** 2023-02-02

**Authors:** Judith M. J. Veugen, Suryan L. Dunker, Petra F. G. Wolffs, Paul H. M. Savelkoul, Bjorn Winkens, Frank J. H. M. van den Biggelaar, Rudy M. M. A. Nuijts, Mor M. Dickman

**Affiliations:** * University Eye Clinic, Maastricht University Medical Center+, Maastricht, the Netherlands;; † School for Mental Health and Neuroscience (MHeNs), Maastricht University, Maastricht, the Netherlands;; ‡ Department of Medical Microbiology, Maastricht University Medical Centre+, Nutrim School of Nutrition and Translational Research in Metabolism, Maastricht, the Netherlands;; § Care and Public Health Research Institute (CAPHRI), Maastricht University, Maastricht, the Netherlands;; ¶ Department of Medical Microbiology and Infection Control, Amsterdam Infection and Immunity Institute, Amsterdam UMC, Vrije Universiteit Amsterdam, Amsterdam, The Netherlands;; ║ Department of Methodology and Statistics, Faculty of Health, Medicine and Life Sciences, Care and Public Health Research Institute (CAPHRI), Maastricht University, Maastricht, the Netherlands; and; ** Department of Ophthalmology, Zuyderland Medical Center, Heerlen, the Netherlands.

**Keywords:** infectious keratitis, corneal transplantation, graft survival

## Abstract

**Purpose:**

The aim of this study was to analyze real-world practice patterns and graft survival after corneal transplantation for infectious keratitis in the Netherlands.

**Methods:**

All consecutive keratoplasties for infectious keratitis registered in the Netherlands Organ Transplant Registry were included. Graft survival was analyzed using Kaplan–Meier survival curves with Cox regression to compare the 3 most common pathogens with subgroup analysis for type and reason of transplantation, sex, and graft size. Multivariable analysis was performed using the same explanatory factors.

**Results:**

Between 2007 and 2017, 1111 keratoplasties for infectious keratitis were registered in the Netherlands Organ Transplant Registry. The most common pathogens were viruses (n = 437), bacteria (n = 271), and *Acanthamoeba* (n = 121). Human leukocyte antigen (HLA) matching did not provide a significant survival benefit, whereas emergency procedures showed worse graft survival [hazard ratio (HR) = 0.40, *P* = 0.120; HR = 2.73, *P* < 0.001, respectively]. Graft size >8.5 mm was significantly worse than graft size 8.5 mm (HR = 2.062, *P* = 0.010). In therapeutic keratoplasty, graft survival was significantly worse for *Acanthamoeba* than viral keratitis (HR = 2.36, *P* = 0.008). In the multivariable model, adjusting for graft size, type, and reason for transplantation, viral and bacterial keratitis did not differ significantly in graft survival, and *Acanthamoeba* showed a significantly worse prognosis (vs. viral keratitis, HR = 2.30, *P* < 0.001; bacterial keratitis, HR = 2.65, *P* < 0.001).

**Conclusions:**

Viral keratitis was the most common indication for transplantation, followed by bacterial and *Acanthamoeba* keratitis. HLA matching did not offer protection over elective non-HLA–matched procedures, whereas emergency procedures and grafts sized >8.5 mm showed poor survival. In optical keratoplasty, survival is high for all pathogens, whereas in therapeutic keratoplasty *Acanthamoeba* shows poor outcome.

Infectious keratitis is a sight-threatening infection that may require corneal transplantation for infection control, visual rehabilitation, and maintaining globe integrity.^1^ In one fourth of countries, it is the leading indication for corneal grafting.^2^ In Europe, corneal transplantation for infectious keratitis is less common, representing 7% of all indications.^3^ Few large studies have reported graft survival of these high-impact procedures.^4,5^ In particular, there are little data on the outcomes of emergency transplants and the role of human leukocyte antigen (HLA) in the context of infectious keratitis.^6^ National quality registries are a valuable tool for improving health outcomes by collecting data from a large number of medical centers.^7^ Registries are therefore poised to assess practice patterns and outcomes of corneal transplantation for infectious keratitis.

In the current study, we analyzed prospectively collected data from the Netherlands Organ Transplant Registry (NOTR) and report the practice pattern and real-world outcomes of corneal transplantation for infectious keratitis in the Netherlands regarding graft survival and the impact of pathogen, type of surgery (HLA matching, emergency, and elective non-HLA–matched procedures), reason for surgery (optical and therapeutic), sex (recipient, donor, and compatibility), and graft size (8.5 mm, <8.5 mm, and >8.5 mm).

## MATERIALS AND METHODS

### Graft Registry and Data Collection

Data for this multicenter prospective registry study were obtained from the NOTR, a prospective national database founded by the Netherlands Transplantation Foundation (Nederlandse Transplantatie Stichting [NTS], https://www.transplantatiestichting.nl/over-de-nts). Donor corneas are centrally allocated and registered in the NOTR in the Netherlands. Using the NOTR, the NTS prospectively captures data related to the recipient, donor, eye bank processing, and surgical procedure of all corneal transplantation performed in the Netherlands. Corneal surgeons completed relevant follow-up data at predefined time points using a standardized electronic data capture system. Data collection was continued until graft failure or loss to follow-up. For this study, the NOTR Steering Group provided institutional review board approval for data extraction and analysis. Informed consent was obtained from all patients to participate in the registry and to use the data for research. The study adhered to the tenets of the Declaration of Helsinki and Dutch legislation.

### Study Population

The first surgery registered in the NOTR was performed on January 2^nd^, 2007. The study cohort included all consecutive corneal transplantation for infectious keratitis, spanning 10 years until December 24^th^, 2017.

### Outcome Measures

The primary outcome measure was graft survival. Secondary outcomes pertain to practice patterns, that is, the volume of transplants and causative agents over time. Graft failure was reported by the corneal surgeon or identified in cases of subsequent corneal transplantation in the same eye. The coding guidelines of the NOTR define primary graft failure as corneal edema that never cleared from the immediate postoperative period. Secondary graft failure is a common end point that can be reached through distinctly different pathways.

### Statistical Analysis

Statistical analyses were performed using the IBM SPSS Statistics for Windows (version 25.0; IBM Corp., Armonk, NY). To avoid dependency between the 2 eyes, repeat transplants were excluded, and for patients who underwent corneal transplantation in both eyes, only the first eye was included in the graft survival analysis. Baseline characteristics are reported as frequencies with percentages or mean ± SD. The number of transplants over time was tested using the χ^2^ goodness-of-fit test. Death-censored graft survival for the total group and subgroups was assessed using Kaplan–Meier survival curves with univariable Cox regression analysis. In addition, a multivariable Cox regression model was performed, including the following explanatory factors: disease-causative agent for the 3 most common pathogens (viral, bacterial, and *Acanthamoeba*), type of surgery (HLA-matched, emergency, or elective non-HLA–matched procedures), reason for surgery (optical keratoplasty performed for visual rehabilitation or therapeutic keratoplasty performed for infectious debulking and preserving globe integrity), and graft size (8.5 mm, <8.5 mm, and >8.5 mm). Cox regression analysis was performed over 5 years or 2 years postoperatively, depending on the number of patients still in the study at that time. The proportional hazard assumption was verified using a log(-log) survival function plot. Two-sided *P* values ≤0.05 were considered statistically significant.

## RESULTS

### Practice Patterns

**FIGURE 1.**
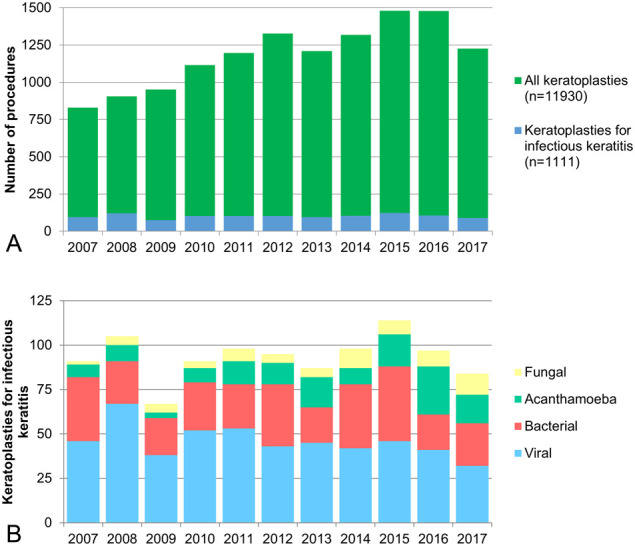
A, Volume of all corneal transplantation (green) compared with corneal transplantation for infectious keratitis (blue) in the Netherlands from 2007 until 2017. The overall volume of corneal transplantation increased over time but remained stable for infectious keratitis. B, Number of transplantation for infectious keratitis per pathogen per year. The number of grafts for *Acanthamoeba* keratitis increased significantly in the second half of the cohort.

In total, 1111 corneal transplant procedures for infectious keratitis were registered in the NOTR between January 2^nd^, 2007, and December 24^th^, 2017. Although the volume of corneal transplant procedures has increased in the last decade, the overall number of transplants for infectious keratitis has not changed significantly over time (*P* = 0.070) (Fig. 1A). Notably, the number of grafts for *Acanthamoeba* keratitis increased significantly in the second half of the cohort (*P* < 0.001) (Fig. 1B). The baseline demographics of all transplants for infectious keratitis are shown in Table 1. For the analysis of graft survival, only primary transplants (n = 829), excluding repeated transplants, were considered. The leading causative agents were viral (n = 437), bacterial (n = 271), and parasitic (ie, *Acanthamoeba*, n = 121). In this cohort of primary transplants, patients were on average 61 (±18) years, 51% (n = 420) were male, 74% (n = 613) were phakic, and 13% (n = 105) were pseudophakic at the time of surgery (1%, n = 7, were aphakic and 13%, n = 104, were missing). Of the surgeries, 65% (n = 540) were optical keratoplasties, 28% (n = 235) were therapeutic keratoplasties, and 7% (n = 54) was missing. HLA matching was performed in 6% (n = 53) of all cases, 15% of all cases (n = 126) were emergency (non-HLA matched) procedures, and 78% (n = 650) were elective non-HLA–matched procedures. The mean graft size was 8.3 mm, 16% of the grafts was 8.5 mm (n = 131), 58% was smaller than 8.5 mm (n = 481), 21% was larger than 8.5 mm, and 5% (n = 44) was missing. For viral, bacterial, and *Acanthamoeba* keratitis, 9% (n = 38), 3% (n = 9), and 5% (n = 6) were HLA-matched; 9% (n = 41), 23% (n = 62), and 15% (n = 18) were emergency (non-HLA matched) procedures; and 77% (n = 335), 69% (n = 184), and 76% (n = 92) were elective non-HLA–matched procedures, respectively. A total of 46 repeated transplants were identified. The causative pathogens included viruses (43%, n = 22), bacteria (15%, n = 7), *Acanthamoeba* (22%, n = 10), and fungi (15%, n = 7). Compared with the incidence of the causative agents of the first transplant, fungal keratitis had the highest risk of repeat transplantation (15%), followed by *Acanthamoeba* keratitis (8%), viral keratitis (5%), and bacterial keratitis (2%), *P* < 0.001. Of repeated transplants, 50% (n = 23) were therapeutic keratoplasties and 50% (n = 23) optical keratoplasties. HLA matching was performed in 11% (n = 5) of repeated transplants, 15% of repeated transplants (n = 7) were emergency procedures, and 74% (n = 34) were elective non-HLA–matched procedures.

**TABLE 1.** Patient, Surgery, and Pathogen Characteristics in All Transplants for Infectious Keratitis

	Mean ± SD or Percentage
Patient age (years)	64 ± 13
Patient sex (male/female)	50%/50%
Lens status (phakic/pseudophakic), n=962	78%/19%
Reason for surgery (optical/therapeutic keratoplasty), n=1026	64%/36%
Type of surgery (HLA matched/emergency procedure/elective non-HLA–matched procedures)	6%/17%/77%
Graft size (mm), n=1058	8.4 ± 1.2
Disease causative agent (virus/bacteria/*Acanthamoeba*/other or unspecified)	46%/28%/13%/14%

N=1111 unless specified otherwise.

### Graft Survival

**FIGURE 2.**
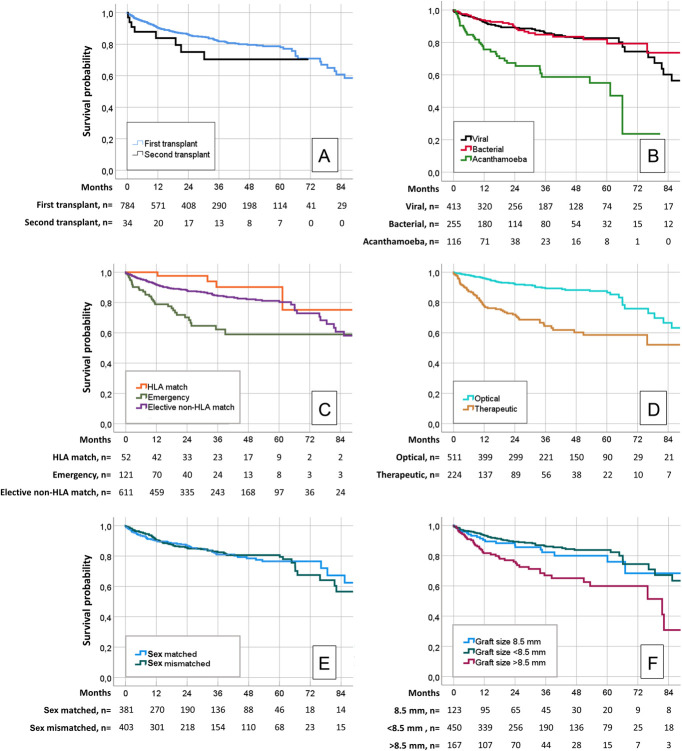
A, Graft survival of primary corneal transplantation and retransplantation for infectious keratitis in the Netherlands. B, Five-year graft survival did not differ between viral and bacterial keratitis but was significantly worse in *Acanthamoeba* keratitis. C, Emergency procedures showed significantly worse graft survival, whereas human leukocyte antigen (HLA) did not provide significant survival benefit compared with elective non-HLA–matched procedures. D, Graft survival of optical and therapeutic keratoplasty in infectious keratitis. In 49 cases, the reason for transplantation was unknown or unclassifiable. HLA = human leukocyte antigen. E, Graft survival did not differ between donor–recipient sex-matched and sex-mismatched cases. F, Five-year graft survival of grafts sized >8.5 mm was significantly worse compared with grafts sized 8.5 mm, whereas grafts sized <8.5 mm did not differ statistically from grafts sized 8.5 mm. In 44 cases, the graft size was unknown.

A total of 784 procedures were available for graft survival analysis. Loss to follow-up was registered in 45 cases (5%). The overall graft survival rates for primary transplantation and retransplantation were 97% and 88% at 3 months, 95% and 88% at 6 months, 91% and 84% at 1 year, 86% and 75% at 2 years, and 82% and 70% at 3 years, respectively (Fig. 2A). The graft survival of primary transplants was 79% at 5 years and 61% at 7 years. No retransplantation reached 5-year follow-up due to failure or censoring. Five-year graft survival did not differ significantly between viral and bacterial keratitis but was significantly worse for *Acanthamoeba* keratitis [compared with viral keratitis, hazard ratio (HR) = 3.37; 95% confidence interval (CI), 2.21–5.15; *P* < 0.001] (Fig. 2B). Graft survival in viral (n = 413), bacterial (n = 255), and *Acanthamoeba* keratitis (n = 116) was 93%, 94%, and 76% at 1 year; 89%, 89%, and 67% at 2 years; 86%, 85%, and 56% at 3 years; and 83%, 79%, and 55% at 5 years, respectively. The outcomes of corneal transplantation for fungal keratitis were analyzed separately due to the low number of cases in our cohort (n = 47) and the high censoring rate (45% of cases available at 1 year). The graft survival for fungal keratitis was 90% (n = 26) at 1 year, 83% (n = 18) at 2 years, 83% (n = 13) at 3 years, and 71% (n = 4) at 5 years.

Graft survival for HLA-matched procedures (n = 52), emergency procedures (n = 121), and elective non-HLA–matched procedures (n = 611) was 100%, 79%, and 92% at 1 year; 98%, 70%, and 88% at 2 years; 91%, 62%, and 85% at 3 years; and 90%, 59%, and 81% at 5 years, respectively (Fig. 2C). Five-year graft survival of HLA-matched grafts did not differ significantly from elective non-HLA–matched procedures (HR = 0.40; 95% CI, 0.13–1.27; *P* = 0.120). Emergency procedures showed significantly worse 5-year graft survival compared with elective non-HLA–matched procedures (HR = 2.73; 95% CI, 1.82–4.09; *P* < 0.001). The graft survival rates for optical (n = 511) and therapeutic keratoplasty (n = 224) were 96% and 78% at 1 year; 92% and 72% at 2 years; 82% and 65% at 3 years; and 88% and 59% at 5 years, respectively (Fig. 2D). In 49 cases, the reason for transplantation was unknown or not classifiable.

No significant differences in 5-year graft survival were observed for both recipient sex (male, n = 392, compared with female, n = 392; HR = 0.97, *P* = 0.857) and donor sex (male, n = 469, compared with female, n = 315; HR = 1.02, *P* = 0.919). Graft survival rates for sex-matched (n = 381) and sex-mismatched (n = 403) cases were 90% and 91% at 1 year; 88% and 86% at 2 years; 83% and 84% at 3 years; and 82% and 83% at 5 years, respectively (Fig. 2E). Five-year graft survival of sex compatible and sex incompatible individuals did not differ significantly (HR = 1.12, *P* = 0.548).

Graft survival for transplants with a graft size of 8.5 mm (n = 123), <8.5 mm (n = 450), and >8.5 mm (n = 167) was 90%, 94%, and 81% at 1 year; 87%, 90%, and 75% at 2 years; 84%, 88%, and 69% at 3 years; and 83%, 87%, and 65% at 5 years, respectively (Fig. 2F). Five-year graft survival of grafts sized <8.5 mm did not differ significantly from grafts sized 8.5 mm (HR = 0.77; 95% CI, 0.45–1.31; *P* = 0.331). However, grafts sized >8.5 mm showed significantly worse 5-year graft survival compared with grafts sized 8.5 mm (HR = 2.06; 95% CI, 1.19–3.58; *P* = 0.010). In 44 cases, the graft size was missing.

**FIGURE 3.**
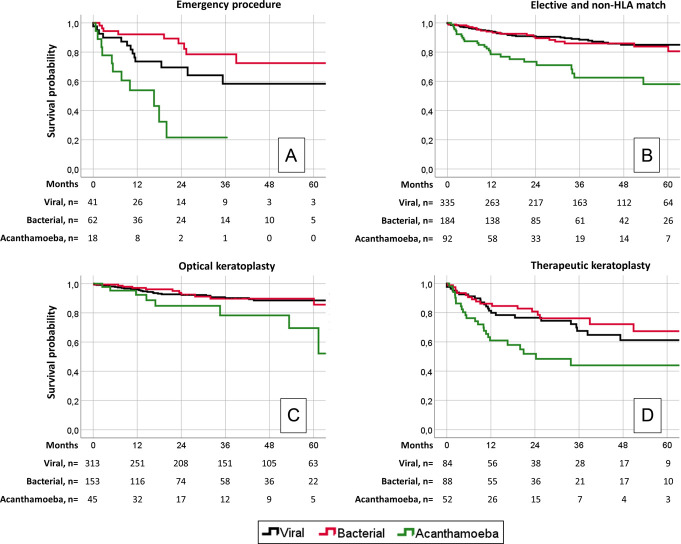
A, In emergency procedures, *Acanthamoeba* keratitis showed significantly worse graft survival compared with viral and bacterial keratitis. B, In elective non-human leukocyte antigen (HLA)-matched procedures, *Acanthamoeba* keratitis showed significantly worse graft survival compared with viral and bacterial keratitis. C, In optical keratoplasty, 2-year graft survival did not differ significantly between pathogens. D, In therapeutic keratoplasty, 2-year graft survival was comparable between viral and bacterial keratitis and significantly worse for *Acanthamoeba* keratitis. HLA = human leukocyte antigen.

In emergency procedures, 2-year graft survival was significantly worse in *Acanthamoeba* keratitis than in viral keratitis (22% vs. 70%, HR = 3.07; 95% CI, 1.32–7.14; *P* = 0.009) and bacterial keratitis (22% vs. 86%; HR = 8.40; 95% CI, 1.60–6.02; *P* < 0.001) (Fig. 3A). In elective non-HLA–matched procedures, 2-year graft survival was significantly worse in *Acanthamoeba* keratitis than in viral keratitis (73% vs. 91%; HR = 3.36; 95% CI, 1.89–5.95; *P* < 0.001) and bacterial keratitis (73% vs. 90%; HR = 3.11; 95% CI, 1.60–6.02; *P* = 0.001) (Fig. 3B).

For optical keratoplasty, 2-year graft survival did not differ significantly between pathogens (*P* > 0.05) (Fig. 3C). For therapeutic keratoplasty, 2-year graft survival was significantly worse in *Acanthamoeba* keratitis than in viral keratitis (52% vs. 77%; HR = 2.36; 95% CI, 1.26–4.46; *P* = 0.008) and bacterial keratitis (52% vs. 81%; HR = 3.02; 95% CI, 1.52–6.02; *P* = 0.002) (Fig. 3D). In a multivariable model, adjusting for type (HLA-matched, emergency, and elective non-HLA–matched procedure) of surgery, reason (optical and therapeutic keratoplasty) for surgery, and graft size, viral and bacterial keratitis did not differ significantly and *Acanthamoeba* showed significantly worse prognosis (Table 2).

**TABLE 2.** Multivariable Analysis of Causative Pathogens for Developing Graft Failure in Infectious Keratitis

Pathogen	Reference	HR	95% CI	*P*
Viral	Bacterial	1.16	0.71–1.88	0.557
*Acanthamoeba*	Viral	2.30	1.41–3.75	<0.001
*Acanthamoeba*	Bacterial	2.65	1.57–4.51	<0.001

Analysis adjusted for type of surgery, reason for surgery, and graft size. CI, confidence interval.

## DISCUSSION

This registry study demonstrated the practice patterns and outcomes of corneal transplantation for infectious keratitis in the Netherlands. Although the volume of corneal transplantation has increased substantially in the last decade due to endothelial keratoplasty, the number of corneal transplants for infectious keratitis has remained stable. Viral, bacterial, and *Acanthamoeba* keratitis were the most common indications. Most transplants were performed for visual rehabilitation (optical keratoplasty), and a minority for debulking and preserving globe integrity (therapeutic keratoplasty). Two-year graft survival was 72% for therapeutic keratoplasty and was comparable between viral and bacterial keratitis but substantially lower in *Acanthamoeba* keratitis, even after correction for type, reason for surgery, and graft size.

Infectious keratitis may rapidly deteriorate, necessitating corneal transplantation in an emergency setting, making it a high-risk therapeutic procedure. In our cohort, emergency procedures resulted in significantly worse graft survival rates. Although information on donor parameters for emergency transplantation is available in the registry, because of the relatively small number of cases compared with nonemergent cases, a comparison can be misleading and was therefore not included. The value of matching HLA subtypes for corneal transplantation remains controversial, and there are little data in the context of infectious keratitis. In the current study, HLA-matched grafts did not provide a significant survival benefit compared with elective non-HLA–matched procedures. Similarly, a recent randomized controlled trial on penetrating keratoplasty for any indication did not find a survival benefit of HLA matching.^8^ However, we do not know what the outcomes would have been, should these high-risk patients have received non-HLA–matched grafts. Nonetheless, it is unlikely that a randomized controlled trial would be conducted in high-risk patients with infectious keratitis, and the registry data may be as close as possible to answer this question. HLA matching in the Netherlands is performed at the discretion of the physician, taking into consideration the characteristics of the recipient and the delay associated with finding a matching donor. Because the use of HLA matching has become rare (<3% according to the EEBA 2008 report), our national registry provides a unique opportunity to explore this topic. A comparison of causative agents within emergency procedures revealed that viral and bacterial keratitis showed promising results, even in so-called high-risk procedures, whereas *Acanthamoeba* showed significantly worse outcomes.

Fungal and *Acanthamoeba* keratitis carried a higher risk of repeat transplantation than viral and bacterial keratitis. Half of the cases involved therapeutic procedures, indicating that the primary graft was insufficient for disease management or did not yield the desired outcome. The Australian Graft Registry reported that a consecutive graft has a lower survival rate than the previous graft.^9^ This trend is confirmed in this study for infectious keratitis and shows the burden on patients and health care.

A much-debated question is the impact of donor–recipient sex matching on graft survival. Previous studies reporting on sex matching in the context of both endothelial keratoplasty and penetrating keratoplasty have shown inconsistent results.^10–13^ Against the background of penetrating keratoplasty for infectious keratitis, we failed to detect a significant effect for donor–recipient matching.

Large-diameter corneal grafts have been previously described as a risk factor for graft failure,^14–16^ but not all studies found a significant association.^17^ Lower graft survival of larger grafts in our cohort may be related to a higher antigenic load in the proximity of the limbus increasing the risk of rejection and a more severe infection requiring debulking.

In most parts of the developed world, viral keratitis is the most common cause of unilateral infectious corneal blindness,^18^ in particular herpes simplex keratitis which affects 1.5 million people globally.^19,20^ In our study, 2-year graft survival for therapeutic viral keratitis measured 77% which is high compared with studies reporting 55%–75% at 2 years.^21–23^ The cause of failure is manifold, for instance, in herpes simplex keratitis, stromal keratitis is associated with deep vascularization which increases rejection risk,^24^ and disease recurrence is common after nerves regenerate in the graft. Moreover, herpes keratitis is accompanied by hypoesthesia, which facilitates recurrent trauma and exposure keratopathy.^25^ The relatively high graft survival in our cohort may be related to standard use of oral prophylaxis to prevent herpes recurrence in the Netherlands.^26^

In bacterial keratitis, the introduction of newer and more potent antibacterial agents led to a decrease in the number of therapeutic keratoplasties in some centers.^27^ Although promising, the US Center for Disease Control estimated that annually 2.8 million people are infected with drug-resistant microbes and resistance is increasing.^28^ In our cohort, the number of keratoplasties varies over time but does not follow a directional trend. Two-year graft survival of therapeutic keratoplasty for bacterial keratitis was 81%, slightly lower than that reported in previous studies (over 90%).^22,29,30^

In our cohort, the volume of corneal transplantation for *Acanthamoeba* keratitis accounted for 14% of all indications and increased from a handful of cases before 2009 to double digits since 2015. This increase may be due to the use of silicone hydrogel contact lenses,^31^ multipurpose solutions,^32^ or local environmental factors.^33^ In line with the literature, corneal grafting for *Acanthamoeba* keratitis showed poor survival outcomes.^30,34–36^ Importantly, *Acanthamoeba* keratitis showed the worst outcomes of all causative agents even when outcomes were adjusted for type of procedure (HLA-matched, emergency, and elective non-HLA–matched procedure), reason for grafting (optical and therapeutic keratoplasty), and graft size. Age was omitted from the multivariable analysis because *Acanthamoeba* predominantly affects younger individuals. Since the introduction of biguanides for medical therapy, most cases are treated medically and therapeutic keratoplasty is traditionally reserved for cases of insufficient response to medical therapy or severe complications.^37^ A much debated question is the value of early therapeutic deep anterior lamellar keratoplasty in *Acanthamoeba* keratitis.^38^ However, at the moment, this is not standard of care in the Netherlands, as a result of which there were too few cases available for separate analyses.

Fungal keratitis has a global incidence of more than 1 million cases each year.^39^ The disease accounts for a significant burden of blindness,^39^ causing nearly 5 times as many corneal perforations as bacterial keratitis.^40^ In our cohort, graft survival for fungal keratitis was 83% 2 years postoperatively, mirroring the outcomes of transplants performed for viral and bacterial keratitis, and comparable to reports in the literature.^41^

Central graft allocation by the NTS ensured the robustness of our primary outcome measure: graft survival. Nonetheless, our study has the following limitations. The NOTR database is not designed to specifically capture differences in virulence, predisposing factors, extensiveness of keratitis, and ocular surface inflammation. Moreover, to reduce the registration burden, not all parameters are mandatory, for example, corneal neovascularization, sutures, and use of topical and systemic immunosuppressive therapy. The time between disease onset and transplantation is also an interesting factor that was unavailable in this study. However, therapeutic and emergency procedures may be used as surrogates.

In conclusion, this prospective Dutch registry study shows that the volume of corneal transplantation for infectious keratitis has remained stable over the last decade, with the exception of *Acanthamoeba*. Viral keratitis was the most common indication, followed by bacterial and *Acanthamoeba* keratitis. Graft survival was promising, except in *Acanthamoeba* keratitis and grafts larger than 8.5 mm. The benefit of HLA matching is unclear, but our data suggest that it may not provide additional survival benefits. Timely therapeutic keratoplasty should be considered as needed given the relatively successful outcomes in our study, especially for viral and bacterial keratitis.

## APPENDIX

The Netherlands Corneal Transplantation Network (excluding writing committee): Lies Remeijer, MD, PhD; Jeroen van Rooij, MD; Robert H.J. Wijdh, MD; Annette J.M. Geerards, MD; Cathrien A. Eggink, MD, PhD; Michel J.W. Zaal, MD, PhD; Carla P. Nieuwendaal, MD; Remco Stoutenbeek, MD, PhD; Tom A. van Goor, MD; Marjolijn C. Bartels, MD, PhD; Bart T.H. van Dooren, MD, PhD; Ype P. Henry, MD; Siamak Nobacht, MD; Mei L. Tang, MD; Robert P.L. Wisse, MD, PhD; Ivanka J. van der Meulen, MD, PhD; Ruth Lapid-Gortzak, MD, PhD; Chantal M. van Luijk, MD; Nathalie T.Y. Santana, MD, PhD; Isabelle Saelens, MD, PhD; Yanny Y. Cheng, MD, PhD; Sacha Gast, MD; Annemiek Rijneveld, MD, PhD; Allegonda van der Lelij, MD, PhD; Michel Zaal, MD, PhD; Nayyirih de Koning Tahzib, MD, PhD; Cesar Sterk, MD; Martine Jager, MD, PhD; Gabriel van Rij, MD, PhD; Mario Dhooge, MD; Hugo van Cleynenbreugel, MD, PhD; Isabel Bleyen, MD, PhD; and Benedicte Putting MD, PhD.

## References

[1] Fine M. Therapeutic keratoplasty. Trans Am Acad Ophthalmol Otolaryngol. 1960;64:786–808. 13699570

[2] Gain P Jullienne R He Z . Global survey of corneal transplantation and eye banking. JAMA Ophthalmol. 2016;134:167–173. 26633035 10.1001/jamaophthalmol.2015.4776

[3] Dunker SL Armitage WJ Armitage M . Practice patterns of corneal transplantation in Europe: first report by the European cornea and Cell transplantation registry. J Cataract Refract Surg. 2021;47:865–869. 33577274 10.1097/j.jcrs.0000000000000574

[4] Shi W Wang T Xie L . Risk factors, clinical features, and outcomes of recurrent fungal keratitis after corneal transplantation. Ophthalmology. 2010;117:890–896. 20079930 10.1016/j.ophtha.2009.10.004

[5] Dunker SL Armitage WJ Armitage M . Outcomes of corneal transplantation in Europe: report by the European cornea and Cell transplantation registry. J Cataract Refract Surg. 2021;47:780–785. 33278237 10.1097/j.jcrs.0000000000000520

[6] Volker-Dieben HJ Kok-van Alphen CC D'Amaro J . The effect of prospective HLA-A and -B matching in 288 penetrating keratoplasties for herpes simplex keratitis. Acta Ophthalmol (Copenh). 1984;62:513–523. 6385606 10.1111/j.1755-3768.1984.tb03962.x

[7] Lauer MS D'Agostino RB. The randomized registry trial—the next disruptive technology in clinical research? N Engl J Med. 2013;369:1579–1581. 23991657 10.1056/NEJMp1310102

[8] Armitage WJ Winton HL Jones MNA . Corneal transplant follow-up study ii: a randomised trial to determine whether HLA class II matching reduces the risk of allograft rejection in penetrating keratoplasty. Br J Ophthalmol. 2022;106:42–46. 33268345 10.1136/bjophthalmol-2020-317543

[9] Williams KKM Galettis R Jones V . The Australian Corneal Graft Registry: 2015 Report. South Australia, Australia: South Australian Health and Medical Research Institute. 2015.

[10] Völker-Dieben HJ Kok-van Alphen CC Lansbergen Q . Different influences on corneal graft survival in 539 transplants. Acta Ophthalmol (Copenh). 1982;60:190–202. 6753453 10.1111/j.1755-3768.1982.tb08373.x

[11] Hopkinson CL Romano V Kaye RA . The influence of donor and recipient gender incompatibility on corneal transplant rejection and failure. Am J Transpl. 2017;17:210–217. 10.1111/ajt.13926 27412098

[12] Price DA Kelley M Price FW Jr. . Five-year graft survival of descemet membrane endothelial keratoplasty (EK) versus descemet stripping EK and the effect of donor sex matching. Ophthalmology. 2018;125:1508–1514. 29731147 10.1016/j.ophtha.2018.03.050

[13] Romano V Parekh M Virgili G . Gender matching did not affect 2-year rejection or failure rates following DSAEK for Fuchs Endothelial Corneal Dystrophy. Am J Ophthalmol. 2022;235:204–210. 34626575 10.1016/j.ajo.2021.09.029

[14] Cherry PM Pashby RC Tadros ML . An analysis of corneal transplantation: I-graft clarity. Ann Ophthalmol. 1979;11:461–469. 378085

[15] Boisjoly HM Bernard PM Dubé I . Effect of factors unrelated to tissue matching on corneal transplant endothelial rejection. Am J Ophthalmol. 1989;107:647–654. 2658619 10.1016/0002-9394(89)90262-6

[16] Li C Zhao GQ Che CY . Effect of corneal graft diameter on therapeutic penetrating keratoplasty for fungal keratitis. Int J Ophthalmol. 2012;5:698–703. 23275903 10.3980/j.issn.2222-3959.2012.06.09 PMC3530811

[17] Bidaut-Garnier M Monnet E Prongué A . Evolution of corneal graft survival over a 30-year period and comparison of surgical techniques: a cohort study. Am J Ophthalmol. 2016;163:59–69. 26706619 10.1016/j.ajo.2015.12.014

[18] Liesegang TJ Melton LJ 3rd Daly PJ . Epidemiology of ocular herpes simplex. incidence in Rochester, MN, 1950 through 1982. Arch Ophthalmol. 1989;107:1155–1159. 2787981 10.1001/archopht.1989.01070020221029

[19] Farooq AV Shukla D. Herpes simplex epithelial and stromal keratitis: an epidemiologic update. Surv Ophthalmol. 2012;57:448–462. 22542912 10.1016/j.survophthal.2012.01.005 PMC3652623

[20] Reynaud C Rousseau A Kaswin G . Persistent impairment of quality of life in patients with herpes simplex keratitis. Ophthalmology. 2017;124:160–169. 27863844 10.1016/j.ophtha.2016.10.001

[21] Limaiem R Mnasri H Merdassi A . Therapeutic penetrating keratoplasty in herpes infected eye. Bull Soc Belge Ophtalmol. 2009;311:37–41. 19621553

[22] Sharma N Sachdev R Jhanji V . Therapeutic keratoplasty for microbial keratitis. Curr Opin Ophthalmol. 2010;21:293–300. 20531191 10.1097/ICU.0b013e32833a8e23

[23] Lomholt JA Baggesen K Ehlers N. Recurrence and rejection rates following corneal transplantation for herpes simplex keratitis. Acta Ophthalmol Scand. 1995;73:29–32. 7627755 10.1111/j.1600-0420.1995.tb00008.x

[24] Kuffova L Knickelbein JE Yu T . High-risk corneal graft rejection in the setting of previous corneal herpes simplex virus (HSV)-1 infection. Invest Ophthalmol Vis Sci. 2016;57:1578–1587. 27050878 10.1167/iovs.15-17894 PMC4824377

[25] Rowe AM Yun HM Hendricks RL. Exposure stress induces reversible corneal graft opacity in recipients with herpes simplex virus-1 infections. Invest Ophthalmol Vis Sci. 2017;58:35–41. 28055100 10.1167/iovs.16-19673 PMC5225994

[26] van Rooij J Rijneveld WJ Remeijer L . Effect of oral acyclovir after penetrating keratoplasty for herpetic keratitis: a placebo-controlled multicenter trial. Ophthalmology. 2003;110:1916–1919. 14522763 10.1016/S0161-6420(03)00798-X

[27] Al-Shehri A Jastaneiah S Wagoner MD. Changing trends in the clinical course and outcome of bacterial keratitis at King Khaled Eye Specialist Hospital. Int Ophthalmol. 2009;29:143–152. 18385946 10.1007/s10792-008-9206-6

[28] Centers for Disease Control and Prevention. Antibiotic Resistance Threats in the United States. Atlanta, GA: Centers for Disease Control and Prevention; 2019. Available from: https://www.cdc.gov/drugresistance/pdf/threats-report/2019-ar-threats-report-508.pdf

[29] Ti SE Scott JA Janardhanan P . Therapeutic keratoplasty for advanced suppurative keratitis. Am J Ophthalmol. 2007;143:755–762. 17335767 10.1016/j.ajo.2007.01.015

[30] Chen WL Wu CY Hu FR . Therapeutic penetrating keratoplasty for microbial keratitis in Taiwan from 1987 to 2001. Am J Ophthalmol. 2004;137:736–743. 15059714 10.1016/j.ajo.2003.11.010

[31] Joslin CE Tu EY Shoff ME . The association of contact lens solution use and Acanthamoeba keratitis. Am J Ophthalmol. 2007;144:169–180. 17588524 10.1016/j.ajo.2007.05.029 PMC2692658

[32] Beattie TK Tomlinson A McFadyen AK . Enhanced attachment of Acanthamoeba to extended-wear silicone hydrogel contact lenses - a new risk factor for infection? Ophthalmology. 2003;110:765–771. 12689900 10.1016/S0161-6420(02)01971-1

[33] Joslin CE Tu EY McMahon TT . Epidemiological characteristics of a Chicago-area acanthamoeba keratitis outbreak. Am J Ophthalmol. 2006;142:212–217. 16876498 10.1016/j.ajo.2006.04.034

[34] Kitzmann AS Goins KM Sutphin JE . Keratoplasty for treatment of Acanthamoeba keratitis. Ophthalmology. 2009;116:864–869. 19410943 10.1016/j.ophtha.2008.12.029

[35] Shi W Liu M Gao H . Perioperative treatment and prognostic factors for penetrating keratoplasty in acanthamoeba keratitis unresponsive to medical treatment. Graefes Arch Clin Exp Ophthalmol. 2009;247:1383–1388. 19424711 10.1007/s00417-009-1103-9

[36] Kashiwabuchi RT de Freitas D Alvarenga LS . Corneal graft survival after therapeutic keratoplasty for acanthamoeba keratitis. Acta Ophthalmol. 2008;86:666–669. 18752517 10.1111/j.1600-0420.2007.01086.x

[37] Dart JK Saw VP Kilvington S. Acanthamoeba keratitis: diagnosis and treatment update 2009. Am J Ophthalmol. 2009;148:487–499 e2. 19660733 10.1016/j.ajo.2009.06.009

[38] Sarnicola E Sarnicola C Sabatino F . Early deep anterior lamellar keratoplasty (DALK) for acanthamoeba keratitis poorly responsive to medical treatment. Cornea. 2016;35:1–5. 26562819 10.1097/ICO.0000000000000681

[39] Brown L Leck AK Gichangi M The global incidence and diagnosis of fungal keratitis. Lancet Infect Dis. 2021;21:E49–E57. 33645500 10.1016/S1473-3099(20)30448-5

[40] Prajna NV Srinivasan M Lalitha P Differences in clinical outcomes in keratitis due to fungus and bacteria. JAMA Ophthalmol. 2013;131:1088–1089. 23929517 10.1001/jamaophthalmol.2013.1612 PMC3845453

[41] Xie L Dong X Shi W. Treatment of fungal keratitis by penetrating keratoplasty. Br J Ophthalmol. 2001;85:1070–1074. 11520759 10.1136/bjo.85.9.1070 PMC1724109

